# Bacteriophage Treatment Induces Phenotype Switching and Alters Antibiotic Resistance of ESBL *Escherichia coli*

**DOI:** 10.3390/antibiotics14010076

**Published:** 2025-01-13

**Authors:** Árpád Rózsa, László Orosz, Nikoletta Szemerédi, Gabriella Spengler, Gábor Kecskeméti, Otília Vágó, Károly Péter Sárvári, Diana Szabó, Zoltán Szabó, Katalin Burián, Dezső Péter Virok

**Affiliations:** 1 Pándy Kálmán County Hospital, Semmelweis Str. 1, H-5700 Gyula, Hungary; rozsa.arpad@bmkk.eu (Á.R.);; 2 Department of Medical Microbiology, Albert Szent-Györgyi Health Center and Albert Szent-Györgyi Medical School, University of Szeged, Semmelweis Str. 6, H-6725 Szeged, Hungaryszemeredi.nikoletta@med.u-szeged.hu (N.S.); spengler.gabriella@med.u-szeged.hu (G.S.); sarvari.karoly.peter@med.u-szeged.hu (K.P.S.); burian.katalin@med.u-szeged.hu (K.B.); 3 Department of Medical Chemistry, Albert Szent-Györgyi Health Center and Albert Szent-Györgyi Medical School, University of Szeged, Dóm Sq. 8, H-6720 Szeged, Hungary; kecskemeti.gabor@med.u-szeged.hu (G.K.); szabo.zoltan@med.u-szeged.hu (Z.S.); 4 Department of Oto-Rhino-Laryngology and Head & Neck Surgery, University of Szeged, Tisza Lajos Str. 111, H-6724 Szeged, Hungary; kunne.szabo.diana@med.u-szeged.hu

**Keywords:** bacteriophage, cocktail, antibiotic, resistance, *Escherichia*, siderophore, metabolism, proteomics, PYOFAG

## Abstract

**Background/Objectives:** Bacteriophage therapy represents a promising strategy to combat multidrug-resistant pathogens, such as *Escherichia coli*. In this study, we explored the effects of a bacteriophage infection on an Extended Spectrum Beta-Lactamase (ESBL) positive *E. coli* isolate. **Methods:** We used next generation sequencing, proteomics and phenotypic screens to investigate the effect of bacteriophage infections on *E. coli* metabolism and resistance phenotypes. **Results:** The bacteriophage infection led to notable alterations in colony morphology, indicating profound changes in bacterial metabolism. Proteomic analysis revealed significant shifts in protein expression, with 65 proteins upregulated and 246 downregulated post-infection. The downregulated proteins were involved in various metabolic pathways, including nucleic acid, protein and lipid metabolism, and iron acquisition. Bacteriophage treatment also led to increased bacterial membrane permeability. Altogether, these alterations in bacterial metabolism and membrane permeability may lead to a general reduction in antibiotic resistance. Indeed, the bacteriophage-infected *E. coli* exhibited increased sensitivity to various classes of antibiotics, including beta-lactams, fluoroquinolones, trimethoprim-sulfamethoxazole, and aminoglycosides. **Conclusions:** Our findings highlight the potential of bacteriophage therapy as an adjunct to existing antibiotics, enhancing their efficacy against resistant strains.

## 1. Introduction

*E. coli*, a member of the *Enterobacteriaceae* family, is a facultative anaerobic bacterium that forms part of the normal microbiota of the gastrointestinal tract. *E. coli* plays an important beneficial role in the colon, such as the competitive exclusion of pathogenic bacteria and the production of vitamin K [1]. However, occasionally, the acquisition of various virulence factors transforms the bacterium into a characteristic pathogenic form. These pathogenic forms are classified into different pathotypes based on the set of virulence factors they possess and the types of diseases they cause [2]. Pathogenic *E. coli* is responsible for gastrointestinal diseases as well as extraintestinal infections, such as meningitis, sepsis, and the most common urinary tract infections. *E. coli* can harbor various antibiotic resistance genes, contributing to challenging treatment scenarios in clinical settings. These antibiotic resistance genes may be located on the bacterial chromosome or on mobile genetic elements like plasmids, which facilitate their transfer between bacteria [3]. *E. coli* antibiotic resistance includes beta-lactam resistance and extended-spectrum beta-lactam resistance, aminoglycoside resistance, fluoroquinolone resistance, and sulfonamide resistance [4]. The impact of antibiotic-resistant *E. coli* infections is significant. Third-generation cephalosporin-resistant and carbapenem-resistant *E. coli* are positioned among the foremost five pathogens on the World Health Organization’s Bacterial Priority Pathogens List 2024, emphasizing the need for the prevention and management of infections caused by antibiotic-resistant pathogens [5]. An alternative to conventional antibiotic therapy for particularly multidrug-resistant bacteria is bacteriophage treatment. Bacteriophages are viruses that replicate within bacteria and either lyse the bacterial host or remain in the host and may lyse it later. Bacteriophage therapy offers several advantages, including a narrow spectrum and high specificity, efficacy against multidrug-resistant strains, reduced side effects and biofilm penetration [6]. Bacteriophage therapy has been applied against *E. coli* in vitro, in animal models, and in humans [7]. Examples of bacteriophage therapy against human *E. coli* infections include urinary tract infections [8], cardiothoracic surgery-related infections [9], recurrent bacteremia, and sepsis [10]. An emerging approach is to use combinations of bacteriophages and antibiotics to achieve a synergistic antimicrobial effect [11]. The benefits of combined treatment include enhanced bacterial growth inhibition, the reduced development of antibiotic/bacteriophage resistance, the reversal of antibiotic resistance, and the improved penetration of antibiotics through biofilms. The improved antimicrobial effect of phage–antibiotic combinations against *E. coli* has been observed before, such as in the case of ECA2 phage and ciprofloxacin [12] and phage EcSw in combination with kanamycin and chloramphenicol [13].

Here, we demonstrate that the use of a bacteriophage cocktail alone significantly affected the proteome and phenotype of an ESBL *E. coli* clinical isolate. Furthermore, we showed a sensitizing effect of bacteriophage to various antibiotics used after bacteriophage treatment.

## 2. Results

### 2.1. Bacteriophage Infection Alters the Growth Kinetics and Colony Morphology of E. coli

**Figure 1:**
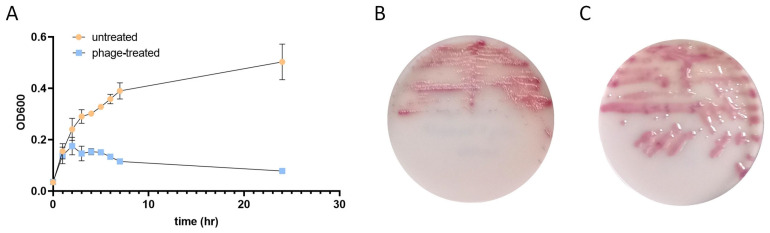
Impact of bacteriophage infection on the growth and colony morphology of *E. coli*: (**A**) Impact of the bacteriophage infection on the growth of *E. coli*. Bacteriophage-treated and untreated *E. coli* were incubated for 24 h at 37 °C with 5% CO_2_. OD_600_ values were measured throughout the experiment. (**B**) Colony morphology of untreated *E. coli*. (**C**) Colony morphology of bacteriophage-treated *E. coli*. At the end of the 24 h incubation period, bacteriophage-treated and untreated *E. coli* were streaked on UriSelect agar and incubated for an additional 24 h at 37 °C with 5% CO_2_.

Approximately 3.2 × 10^6^ *E. coli* (80 µL) was incubated with a PYOFAG cocktail (20 µL) or left untreated for 1 h, 2 h, 3 h, 4 h, 5 h, 6 h, 7 h, and 24 h at 37 °C, 5% CO_2_ (Figure 1A). It is important to acknowledge that the precise concentrations of distinct bacteriophages present in the PYOFAG cocktail remain unspecified; however, as per the manufacturer’s assertions, the concentration exceeds 10^5^ phage particles per milliliter. The untreated *E. coli* concentration exhibited a steady increase, reaching an average OD_600_ value of 0.507 at the 24 h timepoint. Bacteriophage-treated *E. coli* showed similar growth at the 1 h timepoint as the untreated but began to diverge at 2 h post-incubation. Bacteriophage-treated *E. coli* reached its maximum concentration at the 2 h post-infection timepoint and steadily decreased at the later timepoints, achieving an average OD_600_ value of 0.078 at 24 h post-incubation, which is 15.4% of the untreated. After 24 h of incubation, the bacteria were plated onto the UriSelect agar, and the colony morphology of the overnight cultures was evaluated. The untreated *E. coli* displayed characteristic small, pink colonies, while the bacteriophage-treated colonies were large and mucoid (Figure 1B,C).

**Figure 2:**
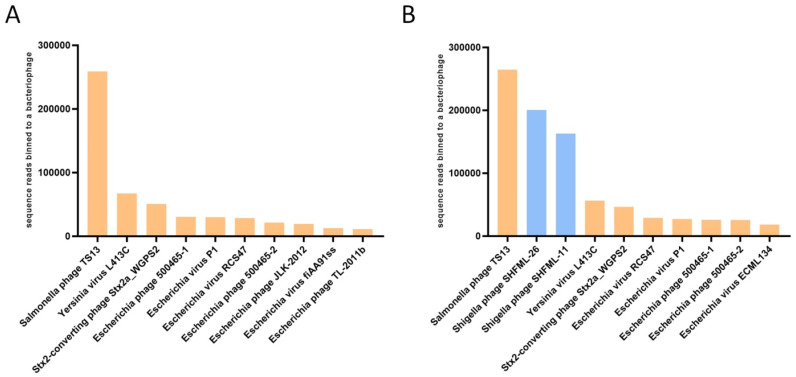
Identification of bacteriophages that infected the *E. coli*: (**A**) Viral sequences identified by whole genome sequencing of the untreated *E. coli*. (**B**) Viral sequences identified by whole genome sequencing of the bacteriophage-treated *E. coli*. Blue columns represent unique bacteriophages in the infected cells.

We applied Illumina whole-genome sequencing of untreated and bacteriophage-infected *E. coli*. Kraken sequence analysis revealed that the untreated *E. coli* genome contained multiple bacteriophage sequences (Figure 2A). The most abundant sequences were associated with the *Salmonella* phage TS13, followed by the *Yersinia* virus L413C and various *Escherichia* phages. In the bacteriophage-treated *E. coli*, these prevalent phage sequences remained, but unique sequences corresponding to *Shigella* phage SHFML-11 and *Shigella* phage SHFML-26 were also significantly present (Figure 2B). These bacteriophages belong to the *Caudoviricetes* class of tailed bacteriophages, featuring an approximately 170 kb long double-stranded DNA genome classified as Baltimore group I [14,15]. PhageScope [16] genome analysis of the previously described SHFML-11 and SHFML-26 phages [14,15] identified 269 and 272 genes, including 55 and 51 hypothetical genes, respectively. PhageScope analysis did not identify any virulence-related genes or antibiotic resistance genes.

### 2.2. Impact of Bacteriophage Infection on the Antimicrobial Resistance of E. coli

**Figure 3:**
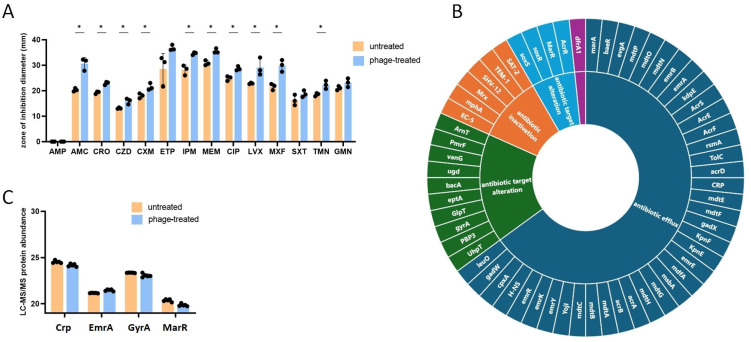
Impact of bacteriophage treatment on the antibiotic sensitivity of *E. coli*: (**A**) Antibiotic sensitivity (diameters of zones of inhibition) of bacteriophage-treated (24 h, 37 °C, 5% CO_2_) and untreated *E. coli* was assessed using the disk diffusion method. Statistical analysis was performed by multiple unpaired t-tests. FDR q values are presented. *: q < 0.05. (**B**) Antibiotic resistance genes identified through whole genome sequencing and the CARD antibiotic resistance gene identification [18] of untreated and bacteriophage-treated *E. coli.* (**C**) Antibiotic resistance proteins with significantly (FDR q value < 0.05) altered expressions following bacteriophage treatment. Protein abundances were quantified by LC-MS/MS (n = 5).

The antimicrobial susceptibility of 24 h PYOFAG-treated *E. coli* was assessed according to the EUCAST guidelines [17] and compared to the untreated control (Figure 3A). Untreated *E. coli* showed resistance to ampicillin (AMP) and cephalosporins (CRO, CZD, CXM) but was sensitive to carbapenems (ETP, IPM, MEM), aminoglycosides (GMN, TMN), and trimethoprim-sulfamethoxazole (SXT). Untreated *E. coli* showed intermediate sensitivity to amoxicillin–clavulanic acid (AMC) and to fluoroquinolones (CIP, LVX, MXF). Since the isolate showed resistance to all of the tested cephalosporins (CRO, CXM, CZD), a test for ESBL production was performed. The ESBL detection test confirmed that the isolate produced ESBL. Interestingly, bacteriophage treatment significantly increased the sensitivity (diameter of zone of inhibition) of 10 of the 14 tested antibiotics related to cell wall synthesis inhibitors, nucleic acid synthesis inhibitors, and protein synthesis inhibitors. This increase in sensitivity was enough to reach the sensitivity breakpoints for CRO, CXM (but not CZD) and CIP, LVX, and MXF. The ESBL disk diffusion test still showed ESBL production after bacteriophage treatment. We used the CARD software (https://card.mcmaster.ca/) [18] to assess whether PYOFAG treatment altered the presence of antibiotic resistance genes (ARGs) in the *E. coli* genome. The CARD screening revealed that untreated *E. coli* harbored 60 ARGs associated with various antibiotic resistance mechanisms and classes (Figure 3B). Most of the ARGs were linked to antibiotic efflux, including the antimicrobial resistance (AMR) gene families: ATP-binding cassette (ABC) antibiotic efflux pumps (five genes), major facilitator superfamily (MFS) antibiotic efflux pumps (fourteen genes), resistance-nodulation-cell division (RND) antibiotic efflux pumps (twenty genes), and small multidrug resistance (SMR) antibiotic efflux pumps (four genes). Six ARGs were associated with antibiotic inactivation, including the EC-5, TEM-1 beta-lactamases, and the ESBL SHV-12. Significant host gene loss induced by bacteriophages is not uncommon, as demonstrated in the case of *E. coli* [19,20]. Notably, sequencing and CARD analysis of the bacteriophage treated *E. coli* did not show any changes to the antibiotic resistance gene repertoire; thus, the observed alteration in resistance phenotypes could not be attributed to the deletion of one or more antibiotic resistance genes. Bacteriophage infection also can alter the expression of antibiotic resistance proteins. LC-MS/MS could detect four proteins with significantly altered expressions (Figure 3C). CrP, GyrA, and MarR protein abundances were decreased after bacteriophage infection, while EmrA expression was increased. According to CARD [18], CrP is a negative regulator of the MdtEF efflux pump expression, which is responsible for resistance against various antibiotics, including fluoroquinolones and macrolides. GyrA is a DNA gyrase, a target of fluoroquinolones, while EmrA is an efflux pump specific for various antibiotics including fluoroquinolones. MarR is a repressor of the *marRAB* operon, which is responsible for the expression of ARGs involved in resistance against beta-lactams, fluoroquinolones, and aminoglycosides. Altogether, the expression of CrP, MarR, and EmrA proteins changed to a direction that could lead to increased antibiotic resistance, while the decreased expression of GyrA may lead to increased sensitivity to fluoroquinolones. Altogether, the limited changes in the number and abundance of resistance-related proteins may not explain the observed general increase in antibiotic sensitivity.

### 2.3. Proteomic Analysis of Bacteriophage-Treated and Untreated E. coli

**Figure 4:**
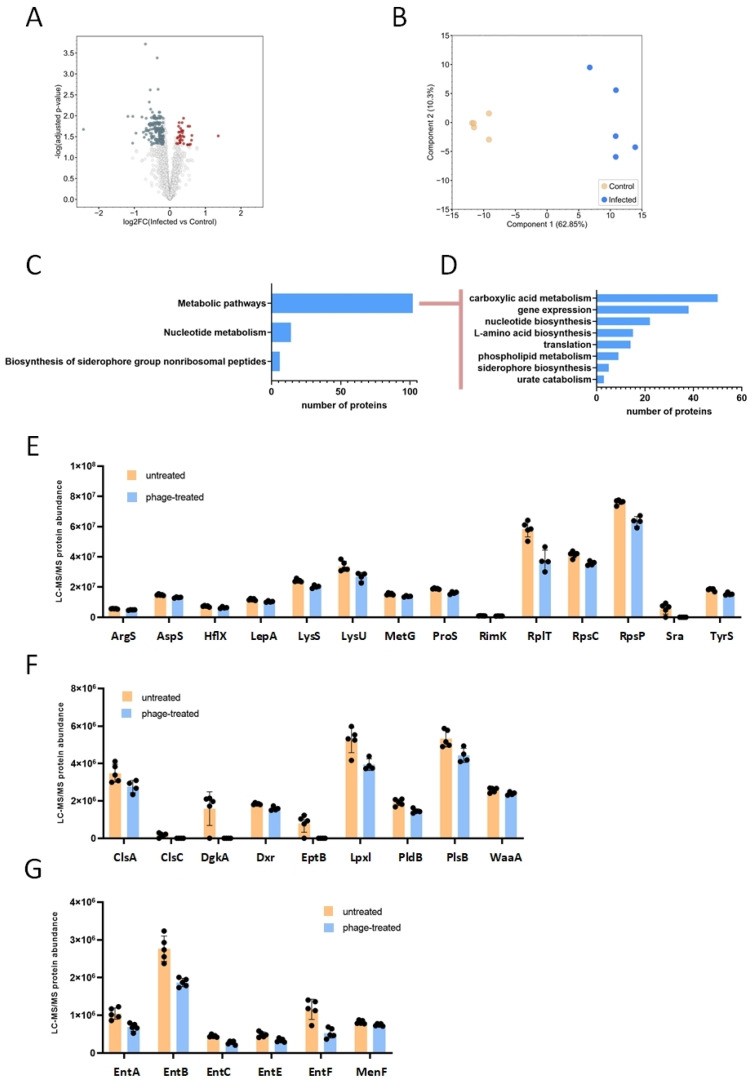
Proteomics of bacteriophage-treated and untreated *E. coli*: (**A**) Volcano plot illustrating the upregulated and downregulated proteins in the bacteriophage-treated (24 h, 37 °C, 5% CO_2_) *E. coli* (bacteriophage-treated/control fold change ≥ 1.14 with q-value < 0.05 (red color); bacteriophage-treated/control fold change ≤ 1/1.14 with q-value < 0.05 (dark gray color)). (**B**) Principal component analysis of the protein abundance data from the five biological replicates of bacteriophage-treated and untreated *E. coli* samples. (**C**) Functional analysis of the downregulated proteins. Significantly enriched (FDR_Benjamini-Hochberg_ q < 0.05) Kyoto Encyclopedia of Genes and Genomes (KEGG) pathways among the downregulated proteins identified by the DAVID database [21]. (**D**) Selected metabolism-related functional groups of the downregulated proteins. (**E**) Downregulated proteins involved in translation. (**F**) Downregulated proteins involved in lipid biosynthesis. (**G**) Downregulated proteins involved in siderophore biosynthesis.

To gain a comprehensive understanding of the phenotypic changes induced by bacteriophages, we used LC-MS/MS to analyze the global protein expression of *E. coli* treated with the bacteriophage cocktail for 24 h. At this timepoint, the bacteriophage infection resulted in the upregulation of 65 proteins while simultaneously downregulating 246 proteins (Figure 4A). The principal component analysis performed on both untreated and bacteriophage-treated *E. coli* samples clearly indicated that the overall protein expression landscape of the bacterium underwent significant alteration post-infection (Figure 4B). The functional analysis of the upregulated proteins did not reveal any significantly enriched cellular pathways. Conversely, the functional analysis of the downregulated proteins indicated their significant association with metabolic processes, nucleotide metabolism, and the synthesis of siderophores (Figure 4C). Among the metabolic processes, various pathways were downregulated by bacteriophage treatment, including carboxylic acid metabolism, nucleotide metabolism, gene expression and translation, lipid metabolism, and siderophore biosynthesis (Figure 4D). Translation-related proteins included ribosomal proteins (RpsP, RpsC, RplT) and several amino acid-tRNA ligases (ArgS, AspS, LysS, LysU, MetG, ProS, TyrS) (Figure 4E). The downregulation of lipid biosynthesis proteins included phospholipid and isoprenoid synthesis (ClsA, ClsC, DgkA, PlsB, LpxL, EptB) and lipopolysaccharide synthesis (WaaA) (Figure 4F). Also, six key proteins involved in siderophore biosynthesis were downregulated (Figure 4G).

### 2.4. Impact of Bacteriophage Treatment on the Membrane Permeability of E. coli

**Figure 5:**
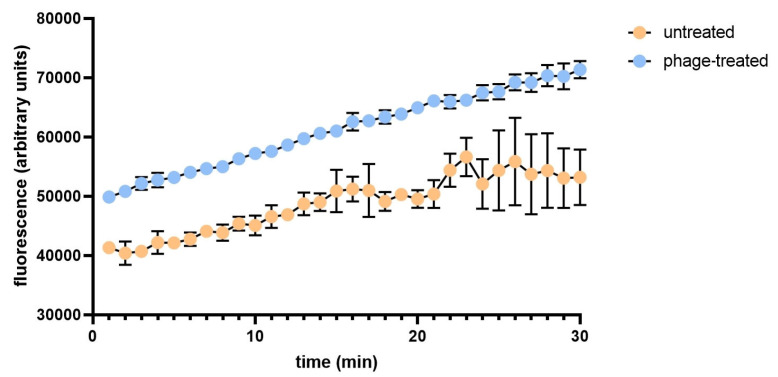
Impact of bacteriophage treatment on the accumulation of ethidium bromide in *E. coli:* Bacteriophage-treated and untreated *E. coli* (n = 3) were incubated for 24 h at 37 °C with 5% CO_2_. After, the bacterial cells were incubated for 30 min in the presence of EB (2 µg/mL). Fluorescence values (λ = 600 nm) were measured throughout the 30 min incubation time.

Proteomic results suggested that changes in metabolism, especially alteration in phospholipid biosynthesis, could lead to increased membrane permeability. To test this hypothesis, we performed an ethidium bromide (EB) accumulation assay of untreated and bacteriophage-treated *E. coli* (Figure 5). At the 0 timepoint, the phage-treated cells had higher fluorescence (average fluorescence intensity 49,878 vs. 42,328), indicating an increased membrane permeability. The difference in EB accumulation increased between the phage-treated and untreated cells by the end of the 30 min incubation time (average fluorescence intensity 71,369 vs. 53,205), indicating an accelerated influx of EB in the phage-infected cells.

## 3. Discussion

Studying the interaction between bacteriophages and antibiotics is important for understanding how to combat antibiotic-resistant bacterial infections. Therefore, we conducted an in vitro study on the interaction between commonly used antibiotics and bacteriophage infections. Our research utilized the commercially available PYOFAG bacteriophage cocktail targeting various Gram-positive and Gram-negative bacteria frequently encountered in clinical practice. DNA sequencing of the untreated and bacteriophage-infected *E. coli* showed that two closely related *Shigella* phages, *Shigella* phage SHFML-26 and SHFML-11, could be detected in the infected bacteria but not in the untreated bacteria. *Shigella* and *Escherichia* genus are closely related; therefore, it is a possibility that there are bacteriophages able to infect both genera [22]. PYOFAG cocktails produced in 1997, 2000, and 2014 have been sequenced previously [23]. A metagenomic analysis of PYOFAG revealed that *i*, there were changes in cocktail composition across the different manufacturing dates; and *ii*, the most recent 2014 cocktail indeed contained a phage named PYO2014_21, which was similar to *Shigella* phage SHFML-11.

While neither SHFML-26 nor SHFML-11 phages contains virulence genes or antibiotic resistance genes, both phages contain more than 50 hypothetical genes. It was shown before that bacteriophage treatment could alter significantly the *E. coli* global gene expression and protein expression [24,25,26]. For example, Wright et al. showed that the φX174 bacteriophage infection of *E. coli* induced the expression of 255 host proteins and repressed 54 [26]. Interestingly, many of the altered host proteins were related to membrane damage and membrane remodeling. The significant changes in bacterial metabolism may lead to phenotype switching. Phenotype switching is a reprogramming of bacterial metabolism, leading to markedly different phenotypic characteristics [27]. Phenotype switching plays an important role in pathogenicity, biofilm formation and antibiotic resistance. A characteristic and easily observable feature of phenotype switching is the change in colony morphology, such as the appearance of small colony variants [28] and mucoid or non-mucoid phenotypes [29]. In our study, the change from the characteristic small, pink colony to mucoid indicated a phenotype switching. To explore the cellular pathways at the background of phenotype switching, LC-MS/MS proteomics was applied. Proteomic analysis revealed a significant change in the global protein expression induced by bacteriophage treatment. Interestingly, among the upregulated proteins, we found two key proteins involved in capsule/biofilm production, the RNA-binding protein HfQ [30], and the DNA binding protein IHF-B [31]. However, altogether, the downregulated proteins were almost four times more numerous than the upregulated ones and impacted various key metabolic pathways. One of the significantly downregulated KEGG pathways was the siderophore biosynthesis. Enterobactin, a siderophore produced by various bacteria, plays a crucial role in iron uptake, which is essential for bacterial growth and survival, particularly in iron-limited environments [32]. Studies indicate a significant correlation between siderophore production and antibiotic resistance in enterococci, particularly with fluoroquinolones. Resistant strains tend to produce higher quantities of siderophores, which may enhance their virulence and complicate treatment [33]. Similarly, in *E. coli*, extensively drug-resistant strains have been linked to siderophore production, suggesting that these mechanisms may co-evolve with resistance traits [34]. Bacteriophage also repressed bacterial translation and lipid biosynthesis, potentially influencing antibiotic resistance. It is well known that protein synthesis inhibitors and cell wall synthesis inhibitors have a synergistic effect against Gram-positive microorganisms [35], and synergistic interactions between protein synthesis inhibitors and nucleic acid synthesis inhibitors have been described for *E. coli* [36]. Protein synthesis inhibition may also impact antibiotic resistance protein expression, and we found four resistance-related proteins with an altered expressions after bacteriophage treatment. However, their resistance action cannot explain the observed general increase in antibiotic susceptibility after bacteriophage treatment. It is possible that other resistance-related proteins were also downregulated by bacteriophage infection, but the LC-MS/MS method was not sensitive enough to detect them. Nine proteins involved in phospholipid biosynthesis, isoprenoid biosynthesis, and lipopolysaccharide biosynthesis were also downregulated by bacteriophage infection. The altered lipid biosynthesis and translation inhibition that may impact outer membrane proteins could lead to increased membrane permeability. Indeed, we could show that bacteriophage infection leads to the increased accumulation of EB, a compound that cannot cross intact plasma membranes. These physiological changes, especially the increased membrane permeability, may lead to a general increase in intracellular antibiotic accumulation. According to this, we observed a general increase in sensitivity to various antibiotics with different cellular targets.

Altogether, our study demonstrated that distinct phenotypes, such as resistance to bacteriophages and antibiotics, can be interrelated. A possible explanation could be that, while bacteria may develop resistance to phages, this can come at a fitness cost, such as increased vulnerability to antibiotics. Analysis of 70 *E. coli* isolates by Allen et al. did not reveal a significant negative correlation between phage resistance and antibiotic resistance; certain phage-antibiotic combinations exhibited a negative, albeit non-significant, tendency [37]. Similarly, *Salmonella enterica* serovar Typhimurium bacteriophage-resistant isolates exhibited a non-significant tendency to show higher sensitivity to various classes of antibiotics and a significantly higher sensitivity to ciprofloxacin [38]. However, in our study, the bacterium was not resistant to phage infection, as we detected two novel bacteriophages in the DNA of the culturable host; rather, it was resistant to bacteriophage-mediated lysis. Our theory is that the non-lytic phage infection altered the proteome that ultimately led to a general increase in antibiotic sensitivity.

Our results suggest that, at least in certain cases, bacteriophage therapy followed by antibiotic combinations could be highly effective, and, in clinical settings, it should be tested before therapy. The host genetic background certainly plays a role, but further studies are needed to identify the exact *E. coli* genotype that may show this type of bacteriophage–antibiotic interaction. 

## 4. Materials and Methods

### 4.1. Bacterium Isolate and Reagents

A clinical isolate of ESBL *E. coli*, *E. coli*_USZ25_ obtained during a routine microbiology diagnosis of a urine sample (Department of Medical Microbiology, University of Szeged, Szeged, Hungary) was utilized in this study. The phenotypic confirmation of ESBL positivity was conducted using the AMPC ESBL Detection Set (MAST, Liverpool, UK). Species-level identification was performed with the MALDI Biotyper (Bruker, Billerica, MA, USA). A PYOFAG bacteriophage cocktail (neoprobio care, Kyiv, Ukraine) containing bacteriophages (>10^5^ phage particles/mL) targeting *E. coli*, *Streptococcus pyogenes*, *Staphylococcus aureus*, *Pseudomonas aeruginosa*, *Proteus vulgaris*, and *Proteus mirabilis* was employed. All other reagents were sourced from Sigma (St. Louis, MO, USA), unless otherwise specified.

### 4.2. Antibiotic Susceptibility and Growth Kinetic Tests

The *E. coli* isolate was incubated in an LB broth overnight at 37 °C. The next day, the OD_600_ was measured and adjusted to 0.05. A total of 80 μL of the bacterial suspension (approximately 3.2 × 10^6^ *E*. *coli*) was transferred to a well of a 96-well plate, and 20 µL of the LB broth or 20 μL of the bacteriophage cocktail was added, followed by overnight incubation at 37 °C, 5% CO_2_. The bacterial suspension was then streaked onto the UriSelect 4 agar (BioRad, Hercules, CA, USA) and incubated overnight at 37 °C, 5% CO_2_. On the following day, isolated colonies were used for antibiotic susceptibility testing. The antibiotic susceptibility of the *E. coli* isolate to ampicillin (AMP, 20 µg), amoxicillin–clavulanic acid (AMC, 20–10 µg), cefuroxime (CXM, 30 µg), ceftriaxone (CRO, 30 µg), ceftazidime (CZD, 10 µg), imipenem (IPM, 10 µg), meropenem (MEM, 10 µg), ertapenem (ETP, 10 µg), levofloxacin (LVX, 5 µg), ciprofloxacin (CIP, 5 µg), moxifloxacin (MXF, 5 µg), tobramycin (TMN, 10 µg), gentamicin (GMN, 10 µg), and trimethoprim-sulfamethoxazole (SXT, 1.25–23.75 µg) was determined using the disk diffusion method according to EUCAST guidelines [18]. For growth kinetic measurements, approximately 3.2 × 10^6^ *E.* *coli* was incubated with the PYOFAG cocktail (20 µL) or left untreated for 1 h, 2 h, 3 h, 4 h, 5 h, 6 h, 7 h, and 24 h at 37 °C, 5% CO_2_. OD_600_ values were measured by an EZ Read 400 (Biochrom, Cambridge, UK) spectrophotometer.

### 4.3. Genome Sequencing of Bacteriophage-Treated and Untreated E. coli

Isolated colonies of bacteriophage-treated (24 h, 37 °C, 5% CO_2_) and untreated *E. coli* were used for genome sequencing. Total genomic DNA was extracted from the samples using the QIAamp DNA Mini Kit (Qiagen, Hilden, Germany). DNA concentration was measured with the Qubit dsDNA HS Assay Kit and Qubit 3.0 Fluorometer (Thermo Fisher Scientific, Waltham, MA, USA). For Shotgun DNA-Seq library construction, the NEXTFLEX^®^ Rapid XP DNA-Seq Kit v2 with UDIs (PerkinElmer, Waltham, MA, USA) was applied according to the manufacturer’s protocol. The library quantities were measured using the Quant-it 1× dsDNA HS Assay kit (Thermo Fisher Scientific, Waltham, MA, USA) with the Fluostar Omega (BMG Labtech, Ortenberg, Germany). The fragment size distribution of the libraries was determined by capillary electrophoresis on the LabChip GX Touch HT Nucleic Acid Analyzer (PerkinElmer) on the X-Mark HT chip (CLS144006, PerkinElmer) using the DNA NGS 3k Assay kit (CLS960013, PerkinElmer). Pooled libraries were diluted to 140 pM for 2×150 bp paired-end sequencing with the 300-cycle 10B Reagent Kit on the NovaSeq X Plus Sequencing System (Illumina, San Diego, CA, USA) according to the manufacturer’s protocol. Raw sequenced reads, >5 Gbp per sample, were demultiplexed using NovaSeq Control Software v1.8, while the FastQ Toolkit was applied to trim adapters and bases at the 3′- and 5′-ends with a quality score of less than 30. Reads with a mean quality score of less than 30 and shorter than 100 bp were filtered out. Quality checks of the sequenced reads were performed using the FastQC program v0.12.1. De novo assemblies for all samples were generated using SPAdes software v3.9.0. Taxonomic characterization of all samples was performed using the Dragen Metagenomics Pipeline v3.5.13 (Illumina, San Diego, CA, USA).

### 4.4. Proteome Analysis of Bacteriophage-Treated and Untreated E. coli by Liquid Chromatography with Tandem Mass Spectrometry (LC-MS/MS)

Isolated colonies of bacteriophage-treated (n = 5) and untreated (n = 5) *E. coli*, as described above, were utilized for proteomic studies. Trifluoroacetic acid (TFA) lysis of both bacteriophage-treated and untreated *E. coli* cells was performed by adding 50 μL of 100% TFA to the pellets, heated for 5 min at 55 °C, and then neutralized with a solution of 450 μL 2 M Tris (pH not adjusted). Protein contents were quantified by a BCA Protein Assay at 4-fold dilution using the manufacturer’s acetone precipitation protocol. For digestion, 20 μg proteins were reduced and alkylated (TFA: 9 mM of tris(2-carboxyethyl)phosphine (TCEP) and 40 mM of chloroacetamide (CAA) for 5 min at 95 °C). The samples were diluted with water to a final concentration of 1 M of Tris and 5% TFA. Trypsin was added in a ratio of 1:50 to all samples and incubated overnight at 30 °C. The digestion was halted with a final concentration of 3% formic acid, and the resulting mixture was analyzed using LC-MS/MS. For the LC-MS/MS analysis, a Waters ACQUITY UPLC M-Class system coupled with an Orbitrap Exploris 240 mass spectrometer (Thermo Fisher Scientific, Waltham, MA, USA) was employed. Chromatographic separation was performed on a C18 analytical column with gradient elution. Data acquisition utilized a data-independent acquisition (DIA) approach, and raw data were processed through DIA-NN to create a spectral library and quantify peptides. The analysis involved filtering precursor identifications at a 1% false discovery rate (FDR) and applying differential expression analysis with specific cut-off values for FDR-corrected *p*-values. Normalization and statistical analysis of LC-MS data were performed using the MS-DAP R-package [39]. Data were normalized using the ‘vsn’, while differential expression analysis was performed with a ‘deqms’ algorithm, using an FDR < 0.05 significance threshold. Proteins identified in at least 3 samples with 2 unique peptides were included in the statistical analysis. Functional classification of the significantly altered proteins was conducted using DAVID software [21]. LC-MS/MS data are available in the Supplementary Materials.

### 4.5. Real-Time Ethidium Bromide Accumulation Assay

To assess the intracellular accumulation of the general membrane permeability indicator ethidium bromide (EB) in untreated (n = 3) and bacteriophage-treated (n = 3) *E. coli*, a real-time EB assay was performed [40]. The intracellular accumulation of the fluorochrome EB was recorded by the CLARIOstar Plus plate reader (BMG Labtech, Cambridge, UK). The bacterial cultures were incubated at 37 °C in a shaking incubator until they reached an optical density (OD) of 0.6 at 600 nm, after they were washed with phosphate-buffered saline (PBS; pH 7.4), centrifuged, and resuspended in PBS. A 50 μL PBS containing a non-toxic concentration of EB (2 µg/mL) was then pipetted into a 96-well black microtiter plate (Greiner Bio-One Hungary Kft, Mosonmagyaróvár, Hungary), and 50 μL of bacterial suspension (OD_600_ 0.6) was added to each well. The plates were placed into the CLARIOstar plate reader, and the fluorescence was monitored every minute for one hour at excitation and emission wavelengths of 530 nm and 600 nm.

## 5. Conclusions

Our study highlights the significant interaction between bacteriophages and antibiotics, particularly in modulating general antibiotic resistance in *E. coli.* The observed phenotype switching and alterations in protein expression underscore the complex biochemical pathways influenced by bacteriophage treatment. Notably, the downregulation of proteins related to key metabolic processes, including lipid biosynthesis and translation, and increased membrane permeability suggest a mechanism by which bacteriophages can generally enhance the antibiotic susceptibility of certain *E. coli* isolates. These findings provide insights into potential therapeutic strategies that leverage bacteriophages to combat antibiotic-resistant bacterial infections.

## Data Availability

Data supporting the reported results can be found in the Supplementary Materials.

## Supplementary Materials

The following supporting information can be downloaded at https://www.mdpi.com/article/10.3390/antibiotics14010076/s1, LC-MS/MS protein expression measurement of bacteriophage-treated and control *E. coli*.
